# Androgen exposure impairs neutrophil maturation and function within the infected kidney

**DOI:** 10.1128/mbio.03170-23

**Published:** 2024-01-11

**Authors:** Teri N. Hreha, Christina A. Collins, Elisabeth B. Cole, Rachel J. Jin, David A. Hunstad

**Affiliations:** 1 Department of Pediatrics, Washington University School of Medicine, St. Louis, Missouri, USA; 2 Department of Molecular Microbiology, Washington University School of Medicine, St. Louis, Missouri, USA; St. Jude Children's Research Hospital, Memphis, Tennessee, USA

**Keywords:** pyelonephritis, *Escherichia coli*, neutrophils, urinary tract infection, immune response, sex differences

## Abstract

Urinary tract infections (UTIs) in men are uncommon yet carry an increased risk for severe pyelonephritis and other complications. In models of *Escherichia coli* UTI, C3H/HeN mice develop high-titer pyelonephritis (most with renal abscesses) in a testosterone-dependent manner, but the mechanisms underlying this phenotype are unknown. Here, using female mouse models, we show that androgen exposure impairs neutrophil maturation in the upper and lower urinary tract, compounded by a reduction of neutrophil function within the infected kidney, enabling persistent high-titer infection and promoting abscess formation. Following intravesical inoculation with uropathogenic *E. coli* (UPEC), kidneys of androgen-exposed C3H mice showed delayed local pro-inflammatory cytokine responses while robustly recruiting neutrophils. These were enriched for an end-organ-specific population of aged but immature neutrophils (CD49d+, CD101–). Compared to their mature counterparts, these aged immature kidney neutrophils exhibited reduced function *in vitro*, including impaired degranulation and diminished phagocytic activity, while splenic, bone marrow, and bladder neutrophils did not display these alterations. Furthermore, aged immature neutrophils manifested little phagocytic activity within intratubular UPEC communities *in vivo*. Experiments with B6 conditional androgen receptor (AR)-deficient mice indicated rescue of the maturation defect when AR was deleted in myeloid cells. We conclude that the recognized enhancement of UTI severity by androgens is attributable, at least in part, to local impairment of neutrophil maturation in the urinary tract (largely via cell-intrinsic AR signaling) and a kidney-specific reduction in neutrophil antimicrobial capacity.

**IMPORTANCE:**

Although urinary tract infections (UTIs) predominantly occur in women, male UTIs carry an increased risk of morbidity and mortality. Pyelonephritis in androgen-exposed mice features robust neutrophil recruitment and abscess formation, while bacterial load remains consistently high. Here, we demonstrate that during UTI, neutrophils infiltrating the urinary tract of androgen-exposed mice exhibit reduced maturation, and those that have infiltrated the kidney have reduced phagocytic and degranulation functions, limiting their ability to effectively control infection. This work helps to elucidate mechanisms by which androgens enhance UTI susceptibility and severity, illuminating why male patients may be predisposed to severe outcomes of pyelonephritis.

## INTRODUCTION

Urinary tract infections (UTIs) are extremely prevalent, and over 80% are caused by uropathogenic *Escherichia coli* (UPEC). Although a majority of UTIs occur in females, the incidence of UTI in males is higher than in females among young infants (<6 months of age) and also rises in elderly men, due largely to prostate enlargement (1–6). Male UTI is also more complicated than in females, with renal infections carrying increased morbidity and mortality and significantly increasing the risk for hypertension, renal scarring, and chronic kidney disease (1, 7–15).

In mouse models of UTI, C57BL/6 and C3H/HeN females exhibit a more robust cytokine response in the bladder compared to male mice within 24 hours post infection (16, 17), eliciting earlier recruitment of immune cells, which favors UTI resolution (17). Male (or androgenized female) C3H/HeN mice, which feature vesicoureteral reflux (a major risk factor for upper-tract UTI in children [18–20]), develop chronic cystitis and pyelonephritis in a testosterone-dependent manner, marked by neutrophilic renal abscess formation with no reduction in kidney bacterial load through at least 28 days post infection (dpi). Meanwhile, normal females typically resolve infection within 7 days (16, 17, 21), indicating that the robust neutrophil infiltration of the kidney in the androgenized host is insufficient to control infection (16, 17, 22).

Testosterone is generally considered to be immunosuppressive in a variety of diseases (23–26), though males are known to have higher circulating neutrophil counts (27, 28) and enhanced recruitment of neutrophils to sites of infection or injury (29, 30). Both male and female neutrophils and their precursors express high levels of androgen receptor (AR) (31), and androgen exposure is associated with reduced neutrophil chemotactic and phagocytic capacity in both human and preclinical models (29, 32–34).

Neutrophil maturation normally occurs in the bone marrow, with immature neutrophils (CD101– in mice, CD10– in humans) exhibiting incomplete nuclear development and reduced granular content, correlated with reduced granular function (as measured by myeloperoxidase activity) and phagocytic capacity *in vitro* (35, 36). Immature neutrophils prematurely released from the bone marrow can migrate to sites of infection at the same rate as mature neutrophils (37, 38), and their accumulation at inflammatory sites correlates with disease progression (38–40).

Neutrophils may remain in circulation or in tissues beyond their typical lifespan and may act as a first line of defense in organ inflammation (41). In states of inflammation (e.g., lipopolysaccharide [LPS] exposure), aged neutrophils traffic to the inflammatory site faster and are better able to adhere to tissue endothelia and to phagocytose bacteria than younger neutrophils (42–44). However, the accumulation of aged neutrophils in tissues can promote further inflammation and tissue damage (45–47).

Here, we demonstrate that the severe pyelonephritis observed in androgen-exposed C3H/HeN mice features the accumulation of a distinct tissue-specific population of neutrophils that are aged but remain immature. Although these aged immature neutrophils properly traffic to foci of bacterial infection in the kidney, they exhibit reduced degranulation and phagocytic capacity, rendering them less effective in controlling infection. We further demonstrate that the maturation failure of neutrophils within the kidney is largely attributable to cell-intrinsic AR signaling. Our findings reveal a novel cellular mechanism by which androgen exposure may predispose to severe pyelonephritis and renal abscess formation.

## RESULTS

### Persistent, high-titer pyelonephritis in androgenized mice is characterized by continuous neutrophil recruitment

**Fig 1:**
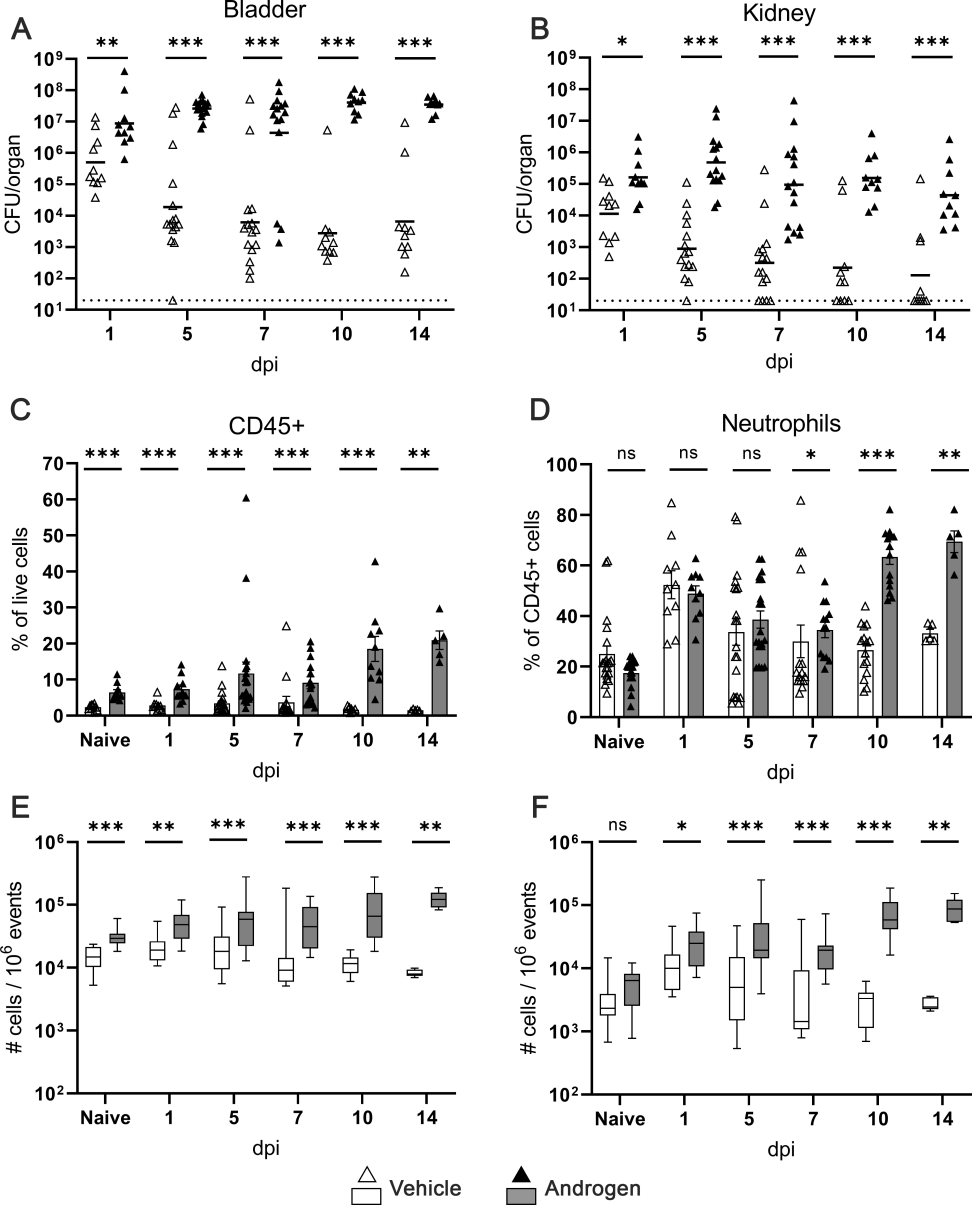
Androgen exposure enables chronic UTI with continuous neutrophil recruitment. (**A and B**) Timeline of bladder and kidney bacterial loads after UPEC inoculation in vehicle-treated (open triangles) and androgenized (filled triangles) C3H/HeN mice. Lines indicate geometric mean. (**C**) CD45+ cell recruitment to the kidney over time as a percentage of live cells, by flow cytometry. (**D**) Neutrophil (CD45+, Ly6G+) recruitment to the kidney over time as a percentage of CD45+ cells. (**E and F**) Timeline of the number of CD45+ cells (**E**) and neutrophils (**F**) in the kidneys throughout the course of infection, expressed per 1 million events, in vehicle-treated (white boxes) or androgenized mice (gray boxes). Bars indicate the mean with SEM. Each symbol represents a single mouse; *n* = 5–15 per condition. *P < 0.05, **P < 0.01, ***P < 0.001 by Mann-Whitney U test. CFU, colony-forming units. ns, not significant.

To mechanistically investigate the effect of testosterone on the neutrophil response to pyelonephritis, we exposed C3H/HeN females to androgen via injection of testosterone cypionate, yielding serum testosterone levels approximating the biological range of adult C3H/HeN males (48), prior to UPEC inoculation. Concordant with our prior results in male and testosterone pellet-implanted female C3H/HeN mice (16, 21) and in testosterone cypionate-treated C57BL/6 females (22, 49), androgenized C3H/HeN females developed chronic cystitis (Fig. 1A) and unresolving pyelonephritis (Fig. 1B), with persistently high bacterial loads across all measured time points. Androgen exposure resulted in increased CD45+ cells in the kidney (as a percentage of live cells and in absolute number) prior to initiation of experimental UTI (i.e., in naïve mice), and CD45+ cell populations increased within the kidneys of androgenized mice as infection progressed (Fig. 1C and E). By 14 dpi, androgenized mice harbored significantly more CD45+ cells in the kidneys than at 1 dpi (*P* = 0.003), while vehicle-treated females had significantly fewer than at 1 dpi (*P* = 0.007; Fig. 1E). By 10 dpi, neutrophils made up ~60% of the CD45+ population in the kidney of androgenized mice, significantly more than that in vehicle-treated mice (Fig. 1D and F). This influx of neutrophils to the kidney occurred before any measurable increase in neutrophils in the peripheral blood (as a proportion of CD45+ cells; Fig. S1A), indicating that neutrophils released from bone marrow were homing to the site of infection. Indeed, vehicle-treated and androgenized mice exhibited similar neutrophil counts in peripheral blood 10 dpi (Fig. S1B). While neutrophil recruitment to the kidney in vehicle-treated mice peaked 1 dpi, this process continued unabated in androgenized mice, with significantly more neutrophils present 14 dpi than at 1 dpi (*P* = 0.003) (Fig. 1F). Among other CD45+ cell types, only T cells increased significantly between 1 and 14 dpi in the kidneys of androgenized mice (Fig. S1C through H).

### Cytokine responses to UTI are intact in the androgenized kidney

**Fig 2:**
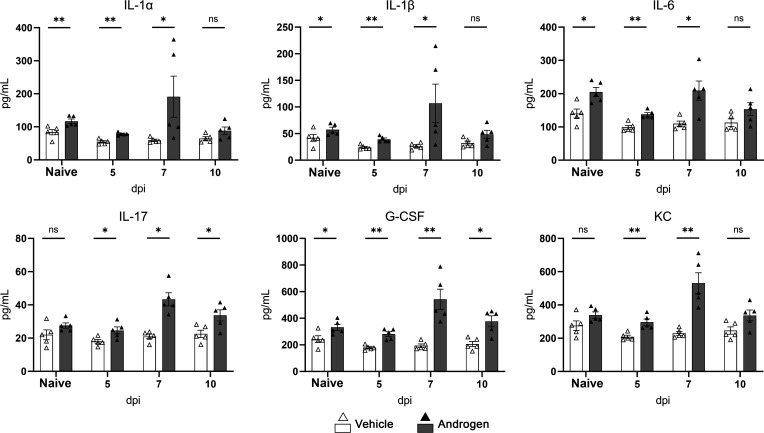
Production of neutrophil recruiting cytokines in the kidney is intact in androgenized mice. Timeline of whole-kidney levels of the indicated cytokines in vehicle-treated (white bars, open triangles) and androgenized mice (gray bars, filled triangles). Bars indicate the mean with SEM. Each symbol represents a single mouse; *n* = 5 per time point. *P < 0.05, **P < 0.01 by Mann-Whitney U test. ns, not significant.

We previously reported that male and androgenized female mice exhibit elevated expression of pro-fibrotic and pro-inflammatory mediators, “priming” the mouse for an aberrant response to UTI (16, 49). This effect was recapitulated in the kidney cytokine profile of androgenized C3H/HeN females, which featured significantly higher levels of G-CSF, IL-1α, IL-1β, and IL-6 than vehicle-treated females prior to UTI (naïve; Fig. 2). Following initiation of UTI, there were no increases in these cytokines, or in IL-17 or KC (CXCL1), measured in the kidneys of vehicle-treated mice (Fig. 2), likely reflecting that whole-kidney cytokine analysis is insensitive to changes associated with modest and localized infection. In androgenized mice 7 dpi, whole-kidney levels of the neutrophil-recruiting cytokines IL-1α, IL-1β, and IL-6 were unchanged from baseline (naïve) (*P* = 0.999, 0.548, 0.999, respectively), while IL-17, G-CSF, and KC (CXCL1) were significantly increased (Fig. 2; *P* = 0.008, 0.016, 0.016, respectively); notably, at this time point, renal abscess is already established (21). Whole-kidney levels of other neutrophil-recruiting (IL-3, GM-CSF) or inhibitory cytokines (IL-4, IL-10) were not significantly altered by androgen exposure at measured time points after infection (data not shown).

### Androgenized mice harbor a distinctly large population of aged, immature neutrophils in infected kidneys

**Fig 3:**
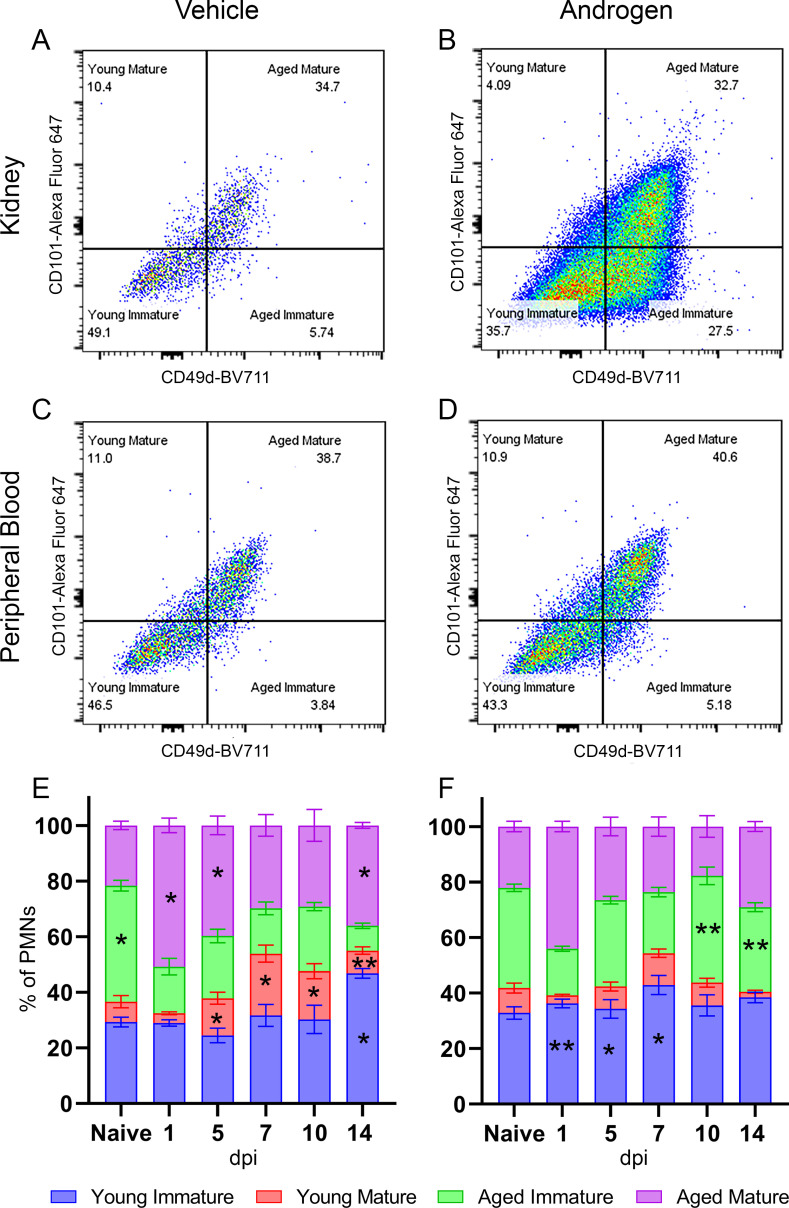
UPEC-infected kidneys in androgenized mice harbor a distinct population of aged immature neutrophils. Representative gating of neutrophils (CD45+, Ly6G+) for age (CD49d) and maturity (CD101) in kidneys (**A and B**) or peripheral blood (**C and D**) from vehicle-treated and androgenized mice 14 dpi. (**E and F**) Timeline of the proportion of neutrophils of each subtype (young immature [blue bars], young mature [red], aged immature [green], aged mature [purple]) throughout the course of infection in vehicle-treated and androgenized mice. Bars indicate the mean with SEM. *n* = 5–15 per condition and time point. *P < 0.05, **P < 0.01 by Mann-Whitney U test.

To investigate how high-titer pyelonephritis persists in the androgenized kidney despite robust neutrophil recruitment, we next interrogated the age and maturity of recruited neutrophils. Using selected flow cytometric markers for age (CD49d) and maturity (CD101), we found that most of the neutrophils (CD45+, Ly6G+) in the kidneys or peripheral blood of vehicle-treated mice were either young and immature (CD49d–, CD101–) or aged and mature (CD49d+, CD101+; Fig. 3A and C). While neutrophils in the peripheral blood of androgenized mice aged and matured similarly to those of vehicle-treated mice (Fig. 3D), the kidneys of androgenized mice accumulated a sizable population of neutrophils that were aged but immature (CD49d+, CD101–; Fig. 3B). Analysis of additional neutrophil markers demonstrated that aged or mature neutrophils were more likely to be CD11b^hi^, CD62L–, CXCR2^lo^, CXCR4+, while young or immature neutrophils were CD11b^lo^, CD62L+, CXCR2^hi^, CXCR4– (Fig. S2I through L). As expected, the aged immature population had intermediate expression of all of these markers compared to populations that were strictly gated on either age or maturity. Ly6G+, CD101–, and CD49d+ neutrophils have been previously described in the bone marrow as committed neutrophil precursors (37); however, in our model, these cells were found in the kidney and not in peripheral blood, indicating they are tissue-infiltrated aged neutrophils rather than neutrophil precursors released prematurely from bone marrow.

Vehicle-treated mice exhibited expansion of multiple neutrophil age/maturity subtypes 1 dpi, dominated by swift recruitment of aged mature neutrophils, with a return to baseline by 10 dpi. In contrast, in androgenized mice, the expansion of young immature and aged (both mature and immature) neutrophils persisted through 14 dpi (Fig. S2A through H). Stated another way, the proportion of aged immature neutrophils remained low as infection resolved in vehicle-treated mice (Fig. 3E; Fig. S2C and G) but had risen significantly in androgenized mice by 10–14 dpi (*P* < 0.01; Fig. 3F; Fig. S2C and G). In addition, the proportion of kidney neutrophils that were aged and mature, though equal at baseline, was significantly lower in androgenized mice than in vehicle-treated mice early in infection (1 and 5 dpi; *P* < 0.05), while the proportion of young immature neutrophils was significantly higher in androgenized mice at these time points (*P* < 0.01; Fig. 3E and F; Fig. S2A and D). Furthermore, the proportion of aged mature neutrophils in the kidneys at later time points (10 and 14 dpi) was significantly lower in androgenized mice (*P* < 0.05 and *P* < 0.01; Fig. 3E and F; Fig. S2B). These data indicate a failure of neutrophil maturation in androgenized hosts at both acute and chronic time points within the infected kidney.

### Maturation impairment is driven by AR signaling in myeloid cells

To determine if androgen-mediated impairment of neutrophil maturation was due to cell type-specific AR signaling, we generated mice lacking expression of AR in myeloid cells (including neutrophils; LysM-Cre × AR^f/f^) or renal tubular epithelium (Ksp-Cre × AR^f/f^). Whole-kidney quantitative PCR (qPCR) showed that Ksp-Cre × AR^f/f^ mice had reduced *Ar* expression compared with Cre^–^AR^f/f^ controls, while bone marrow *Ar* expression in LysM-Cre × AR^f/f^ was nearly absent (only one LysM-Cre × AR^f/f^ sample had detectable *Ar* expression) (Fig. S3A). Of note, the genetically modified parent strains exist in the C57BL/6 background, which does not feature the vesicoureteral reflux characteristic of C3H/HeN mice; we previously demonstrated that androgenized C57BL/6 females maintain lower kidney bacterial loads than similarly treated C3H/HeN mice but are susceptible to persistent pyelonephritis and renal scarring (22, 49). Infection of androgenized Ksp-Cre × AR^f/f^ and LysM-Cre × AR^f/f^ mice resulted in high-titer cystitis and pyelonephritis 7 dpi, similar to that of androgenized Cre^–^AR^f/f^ littermate controls (Fig. S3B) and accompanied by a similar influx of neutrophils to the kidney, both as a percentage of CD45+ cells and in absolute number (Fig. S3C and D).

**Fig 4:**
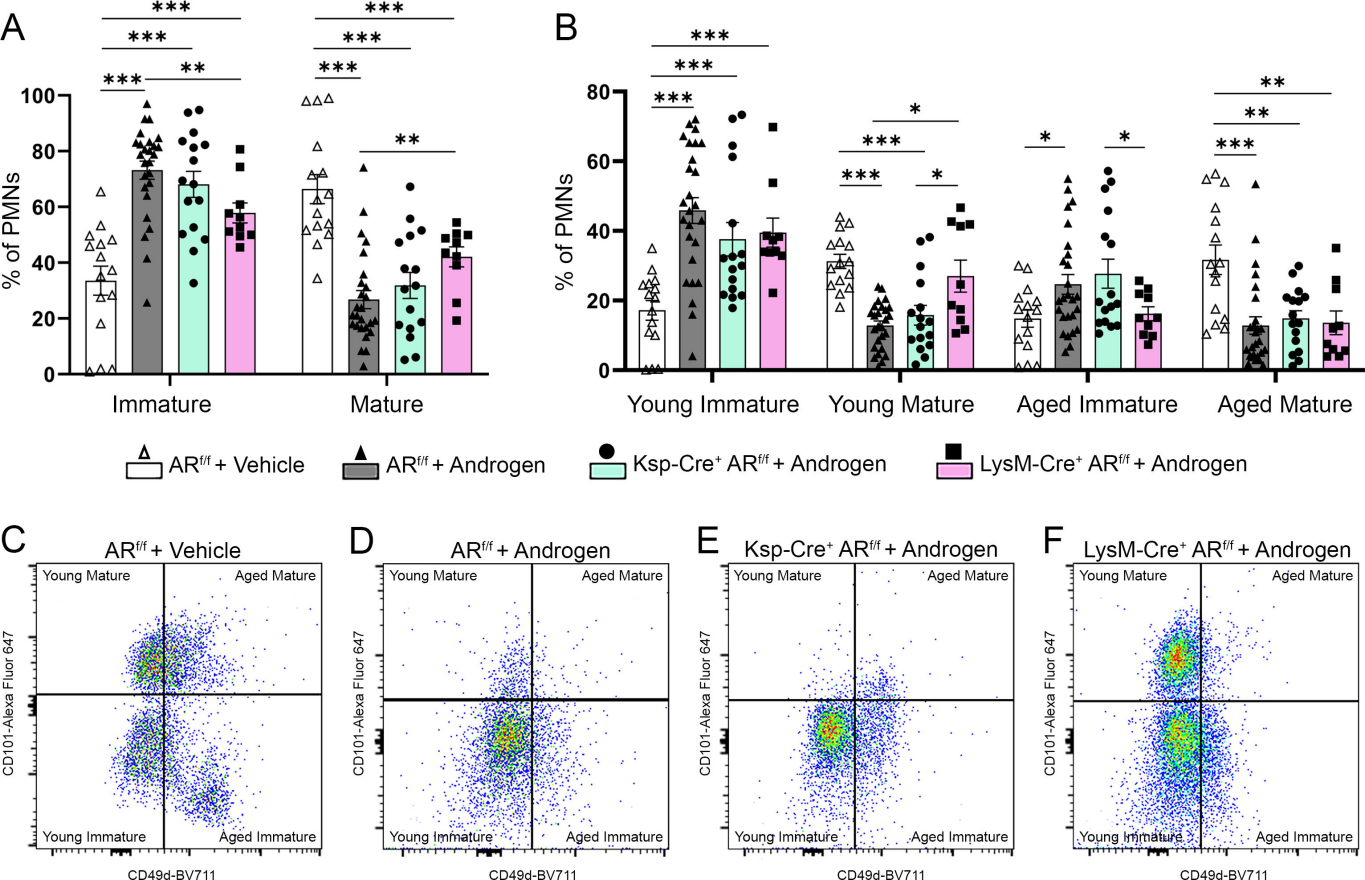
Inhibition of AR signaling in neutrophils substantially restores their maturation in the infected kidney. (**A**) The proportion of neutrophils that were immature (CD101–) or mature (CD101+) 7 dpi in vehicle-treated Cre^–^AR^f/f^ (open triangles, white bars), androgenized Cre^–^AR^f/f^ (closed triangles, gray bars), androgenized Ksp-Cre × AR^f/f^ (squares, green bars), or androgenized LysM-Cre × AR^f/f^ (circles, pink bars) C57BL/6 mice. (**B**) The proportion of neutrophils that were young immature, young mature, aged immature, and aged mature in vehicle-treated Cre^–^AR^f/f^, androgenized Cre^–^AR^f/f^, androgenized Ksp-Cre × AR^f/f^, or androgenized LysM-Cre × AR^f/f^ C57BL/6 mice 7 dpi (symbols as in panel A). Bars indicate the mean with SEM. (**C–F**) Representative pseudocolor plots of neutrophil age (CD49d) and maturity (CD101) by flow cytometry in vehicle-treated Cre^–^AR^f/f^, androgenized Cre^–^AR^f/f^, androgenized Ksp-Cre × AR^f/f^, or androgenized LysM-Cre × AR^f/f^ C57BL/6 mice. *n* = 15–26 per mouse strain. *P < 0.05, **P < 0.01, ***P < 0.001 by Mann-Whitney U test. Comparisons with *P* values >0.05 are not indicated.

Androgen exposure of Cre^–^AR^f/f^ control mice resulted in a higher proportion of immature neutrophils in the kidney 7 dpi compared to vehicle-treated Cre^–^AR^f/f^ littermates (Fig. 4A, C, and D), recapitulating the maturation block observed in C3H/HeN mice. This effect was incompletely but significantly mitigated in androgenized LysM-Cre × AR^f/f^ mice (gray *vs.* pink bars, Fig. 4A). More specifically, compared to androgenized Cre^–^AR^f/f^ control mice, the kidneys of infected LysM-Cre × AR^f/f^ mice displayed a significant decrease in aged immature neutrophils (Fig. 4B). These effects were not seen in Ksp-Cre × AR^f/f^ mice (gray *vs.* green bars, Fig. 4A and B). Overall, neutrophil maturation in the kidney was rescued in these LysM-Cre × AR^f/f^ mice, matching vehicle-treated Cre^–^AR^f/f^ littermates (Fig. 4D and F), while maturation was not rescued in Ksp-Cre × AR^f/f^ mice (Fig. 4D and E). Taken together, these data demonstrate that kidney neutrophil maturation was similarly impaired by androgen exposure in both B6 and C3H models and that the androgen-dependent neutrophil maturation defect is driven substantially by myeloid cell-specific AR signaling. Of note, neutrophil maturity in the kidneys of B6 mice did not correlate with neutrophil age as directly as was seen in C3H/HeN mice. Specifically, while C3H/HeN mice harbored very few young mature neutrophils in the kidneys 7 dpi (Fig. 3A and B), most of the neutrophils in B6 kidneys 7 dpi were young, with fewer aged neutrophils present in the kidneys of all groups (Fig. 4C through F).

### Phagocytosis of UPEC is inhibited in neutrophils of androgenized mice

**Fig 5:**
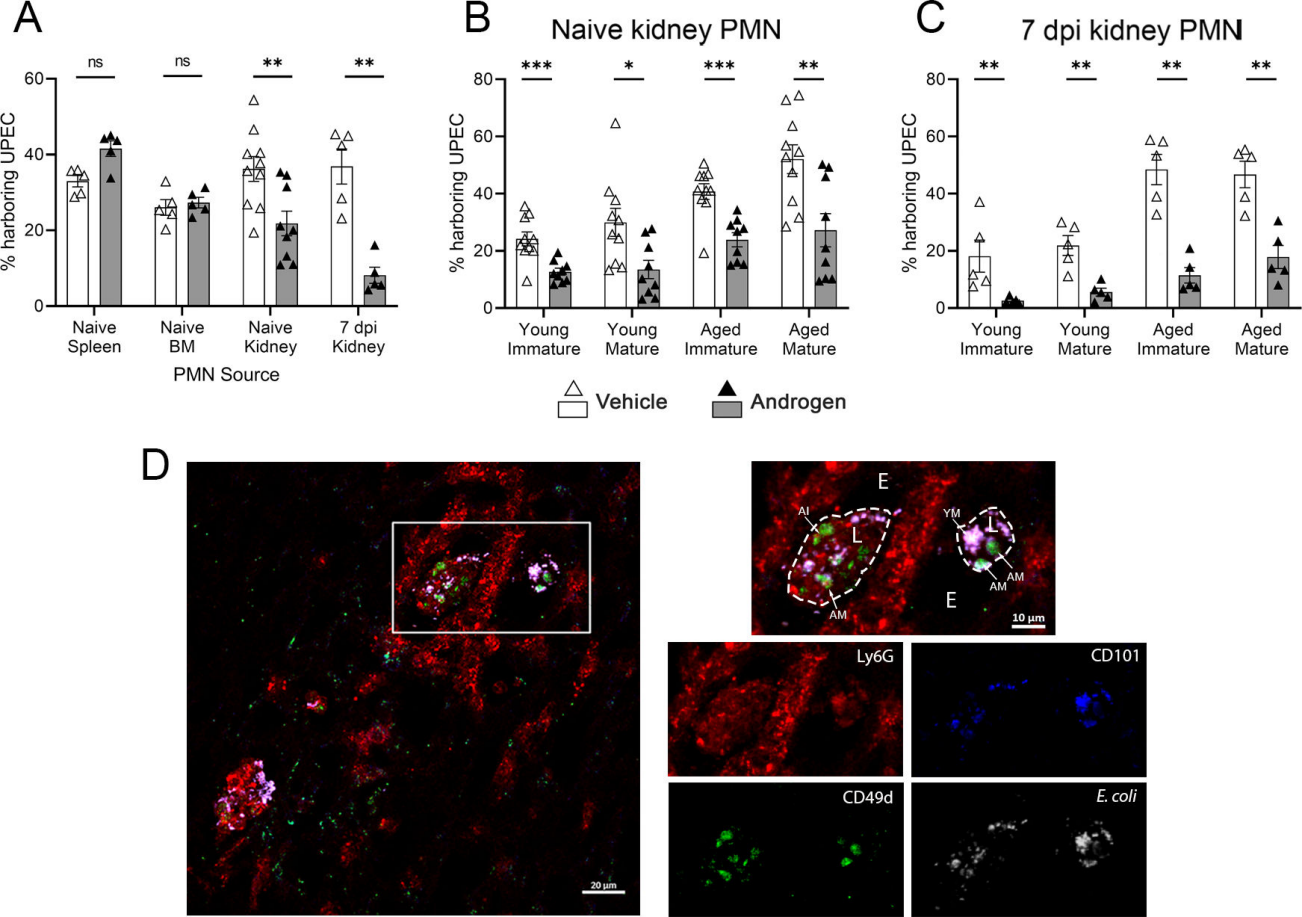
Kidney neutrophils in androgenized mice exhibit reduced phagocytic capacity. (**A**) Percentage of neutrophils that phagocytosed GFP+ UPEC after isolation from the spleen, bone marrow, or kidney in the naïve state, or the kidney 7 dpi, in vehicle-treated (open triangles) or androgenized mice (filled triangles). (**B and C**) Percentage of young immature, young mature, aged immature, and aged mature neutrophils isolated from naïve or 7-dpi kidneys in vehicle-treated or androgenized mice. (**D**) Representative immunofluorescence localization of neutrophil subtypes near and within kidney bacterial communities of an androgenized mouse 10 dpi. White, *E. coli*; red, Ly6G (neutrophils); green, CD49d (age); blue, CD101 (maturity). E, epithelium; L, tubular lumen. Arrows indicate different neutrophil subtypes: YM, young mature; AI, aged immature; AM, aged mature. Bars indicate the mean with SEM. Each symbol represents a single mouse; *n* = 5–10 per group. *P < 0.05, **P < 0.01, ***P < 0.001 by Mann-Whitney U test. ns, not significant.

To further interrogate the effect of androgen exposure on neutrophil functions, leukocytes were isolated from the spleen, bone marrow, or kidneys of vehicle-treated or androgenized mice that had no prior infection (naïve) or were 7 days post UPEC infection. These isolated cells were tested *ex vivo* for their capacity to phagocytose UPEC expressing green fluorescent protein (GFP). Neutrophils (Ly6G+) harvested from any sampled site in vehicle-treated mice had comparable phagocytic capacity, with ~25% to 40% positivity for GFP (white bars, Fig. 5A). Exposure of mice to androgen did not affect phagocytosis by neutrophils isolated from either spleen or bone marrow (Fig. 5A). However, kidney neutrophils from androgenized naïve mice exhibited significantly less phagocytic activity than their vehicle-treated counterparts (~20% GFP+, *P* < 0.01); this phenotype was even more pronounced in kidney neutrophils harvested from androgenized mice 7 dpi (~10% GFP+, *P* = 0.007 vs vehicle) (Fig. 5A). Of note, there remained excess UPEC available for engulfment at the end of incubation in all conditions (Fig. S4A). Kidney neutrophils from androgen-exposed mice were similarly impaired in ingesting heat-killed UPEC (Fig. S4B). Reduced phagocytic capacity associated with androgen exposure was also observed in other (CD45+, Ly6G–) leukocyte populations from the kidney, but not from the spleen (Fig. S4C), indicating that the local environment of the androgen-exposed kidney limits the functional potential of phagocytes, even in the uninfected state.

We next quantified phagocytic capacity of kidney neutrophils according to age/maturity subtypes. Among neutrophils from the kidneys of naïve mice, aged neutrophils exhibited more robust phagocytosis of UPEC than young neutrophils, and mature neutrophils were slightly more effective than their immature counterparts, consistent with prior reports (35, 36, 44), in both vehicle-treated and androgenized groups (Fig. 5B). Furthermore, phagocytic capacity was significantly higher in neutrophils (across all subtypes) from vehicle-treated mice than from androgenized mice (Fig. 5B). These androgen-dependent differences across all subtypes were even more striking among neutrophils from the kidneys of mice infected with UPEC for 7 days (Fig. 5C). Moreover, phagocytic capacity of immature (both young and aged) kidney neutrophils diminished significantly in androgenized mice between the naïve and 7-dpi state (gray bars in Fig. 5B vs 5C, *P* = 0.001 and 0.007, respectively), while there were no differences in phagocytic function of any neutrophil subtype in vehicle-treated mice between the naïve and 7-dpi state (white bars in Fig. 5B vs 5C).

We previously showed that intratubular kidney bacterial communities (KBCs) within the developing renal abscess are surrounded by a large population of neutrophils (16). Here, we used immunofluorescence microscopy to localize kidney neutrophils by age and maturity subtypes in relation to the KBC. As shown before, KBC-bearing tubules were surrounded by Ly6G+ neutrophils; most of these stained as young and immature (Fig. 5D). Neutrophils of each subtype could be found within the KBC itself, suggesting that functional defects in aged immature neutrophils do not include outright failure of trafficking to intratubular UPEC. Consistent with the *ex vivo* results (Fig. 5A through C), *E. coli* staining co-localized exclusively with mature neutrophils (CD101+), with no *E. coli* positivity in aged immature neutrophils (Fig. 5D). This observation suggests that while infiltrating neutrophils (regardless of maturation status) are able to reach UPEC within KBCs, the aged immature neutrophils that are much more prominently represented in the androgenized host exert little phagocytic activity in that niche.

### Degranulation by kidney neutrophils in response to UTI is blunted in androgenized mice

**Fig 6:**
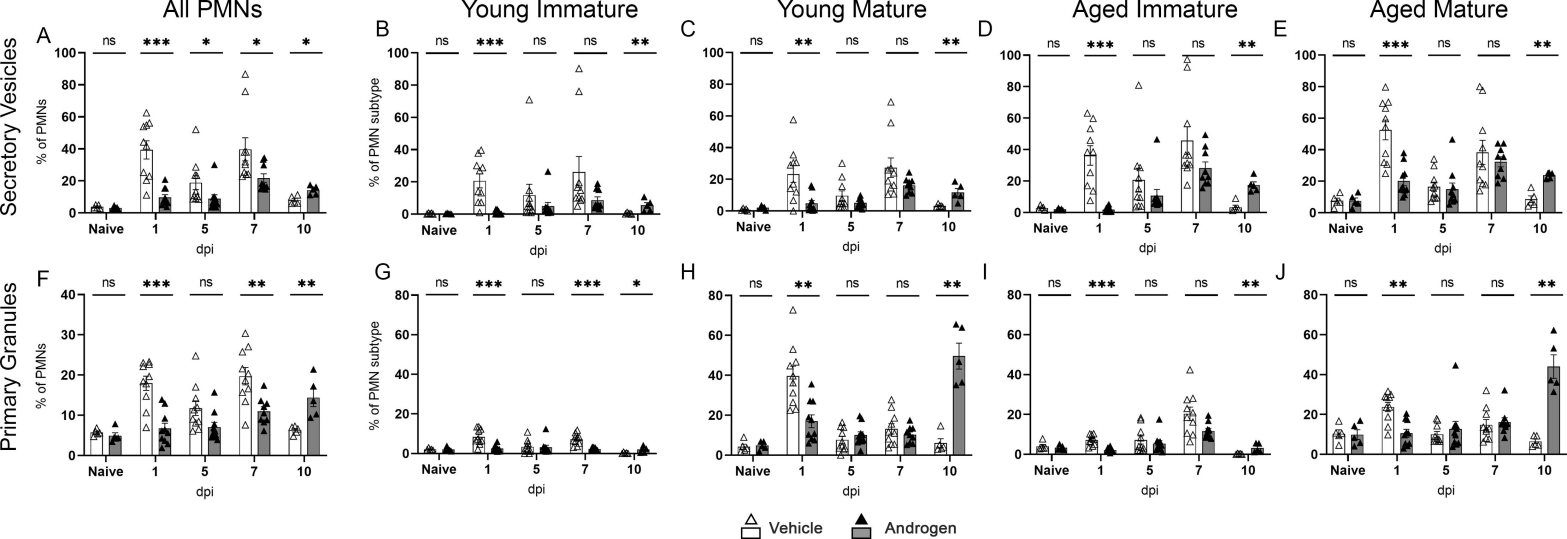
Degranulation by kidney neutrophils is blunted and delayed in androgenized mice. Release of secretory vesicles (CD18+ CD11b+; **A–E**) and primary granules (CD63+; **F–J**), expressed as a proportion of all neutrophils (**A, F**) or of each neutrophil subtype at the indicated time points post UPEC infection in vehicle-treated (open triangles) or androgenized mice (filled triangles). Bars indicate the mean with SEM. Each symbol represents a single mouse; *n* = 5–10 per time point. *P < 0.05, **P < 0.01 by Mann-Whitney U test. ns, not significant.

Neutrophils bear multiple types of granules with specific antimicrobial contents, released in a defined order—secretory vesicles, followed by tertiary (gelatinase) granules, then secondary (specific) granules, and finally primary (azurophilic) granules (50–52). Returning to the C3H/HeN model, we next assessed the extent and tempo of degranulation in kidney neutrophils using flow cytometry markers detectable on the cell surface after selected granules fused with the cell membrane. In vehicle-treated mice, the release of secretory vesicles and primary granules from kidney neutrophils rose 1–7 dpi (compared to the naïve state; Fig. 6A and F), then fell to baseline by 10 dpi in concert with resolving bacterial loads (see Fig. 1A and B). In contrast, degranulation by kidney neutrophils from androgenized mice was blunted 1 dpi and remained lower through 7 dpi (Fig. 6A and F). This suppressed functional response in kidney neutrophils of the androgenized host correlates temporally with failure to control bacterial loads in the tissue (Fig. 1A and B) and formation of abscesses (21). Following this ineffective initial response, the release of primary granules increased by 10 dpi, toward levels observed days earlier in vehicle-treated mice (Fig. 6F). This degranulation at 10 dpi was specifically attributable to the mature neutrophil populations (Fig. 6H and J), consistent with previously published literature showing that immature neutrophils have reduced granular function (35, 50). Indeed, aged immature neutrophils exhibited significantly less degranulation activity at 10 dpi compared to their aged mature counterparts in both vehicle-treated and androgenized mice (*P* = 0.055 for secretory vesicles, 0.008 for primary granules in vehicle-treated mice; *P* = 0.032 for secretory vesicles, 0.008 for primary granules in androgenized mice). As observed earlier, these aged immature neutrophils comprise nearly 40% of the kidney neutrophil population 10 dpi in androgenized mice (*P* < 0.01 vs vehicle-treated; Fig. 3F; Fig. S2C), likely compounding the inability of androgenized mice to effectively clear UPEC from the kidneys.

### Functional defects in neutrophils are specific to the androgenized kidney

**Fig 7:**
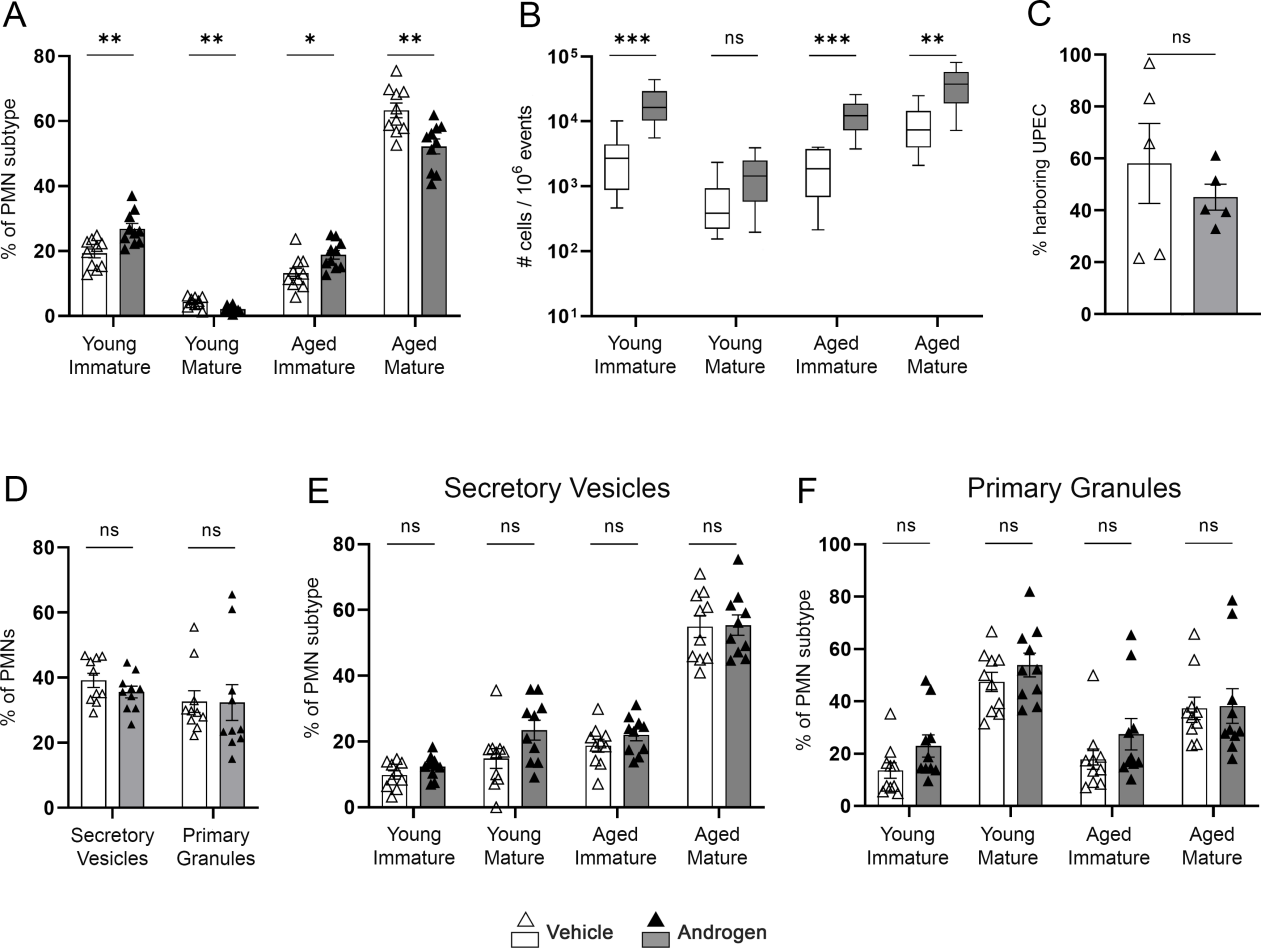
Neutrophils in the UPEC-infected bladder do not share the functional deficits seen in the kidney. (**A**) Distribution of neutrophil age and maturity subtypes in the bladders of vehicle-treated (open triangles, white bars) or androgenized mice (filled triangles, gray bars) 1 dpi as a proportion of total neutrophils. (**B**) Absolute count of each neutrophil age and maturity subtype per million events in the bladder 1 dpi. (**C**) Phagocytosis of GFP+ UPEC by bladder neutrophils harvested 1 dpi. (**D**) The proportion of bladder neutrophils exhibiting degranulation activity 1 dpi. (**E and F**) The proportion of bladder neutrophils 1 dpi from each age and maturity subtype that had released secretory vesicles (**E**) or primary granules (**F**). Each symbol represents a single mouse; *n* = 5–10 per group. **P < 0.01, ***P < 0.001 by Mann–Whitney U-test. ns, not significant.

To further specify whether androgen-dependent defects in neutrophil maturity and function are kidney-specific during UTI, we performed flow cytometry with leukocyte markers on bladder tissue preparations from vehicle-treated and androgenized mice 1 and 5 dpi. Androgenized mice harbored significantly more total CD45+ cells and neutrophils in the bladder 1 dpi (Fig. S5A and B). By 5 dpi, the number of neutrophils in the bladders of both vehicle-treated and androgenized mice had decreased significantly (*P* = 0.019 and *P* = 0.001, respectively; Fig. S5B). Because the absolute cell numbers were much lower in the bladder compared with the kidney, we could only reliably ascertain the age, maturity, and degranulation of the bladder neutrophils 1 dpi. As was observed in the kidney (Fig. S2A through D), the bladders of androgenized mice 1 dpi exhibited a significantly higher proportion of immature neutrophils, and a correspondingly lower proportion of mature neutrophils, compared to vehicle-treated mice (Fig. 7A). Absolute neutrophil numbers were higher in the androgenized bladder across subtypes (Fig. 7B). Strikingly, however, while phagocytic capacity and degranulation were significantly diminished in kidney neutrophils (all subtypes) in androgenized mice (1 dpi, Fig. 5 and 6), those functions of bladder neutrophils isolated 1 dpi were unaltered by androgen exposure (Fig. 7C through F). Taken together, our data indicate that androgen signaling adversely affects neutrophil maturation throughout the urinary tract, while androgen-driven impairment of neutrophil function is observed specifically in the kidney environment.

## DISCUSSION

UTIs are very common in humans, particularly in women (53–56). While UTI in males is less frequent, it is more complicated and severe and can exert long-term negative impacts on renal function (1, 7–15). Androgen-exposed C3H/HeN mice infected with UPEC develop persistent high-titer pyelonephritis accompanied by renal abscesses, comprising large populations of neutrophils surrounding tubules harboring luminal UPEC communities (KBCs) (16, 21). While the kidneys of these mice are subject to an unabating influx of immune cells, predominantly Ly6G+ neutrophils, they show no decrease in bacterial load at any measured time point. We, therefore, sought to determine how and why these recruited neutrophils in androgenized mice fail to effectively control UPEC infection. In this study, we determined that this sex difference in control of renal infection reflects an expanded population of aged immature neutrophils in both bladder and kidneys and a kidney-specific decrement in neutrophil antimicrobial functions in the androgen-exposed host.

Although males have more circulating myeloid cells than females, testosterone is recognized to exert immunosuppressive effects (23–26, 28). Here, we observed similar phenotypes, with androgenized mice harboring more CD45+ cells than vehicle-treated controls in the blood and kidneys prior to initiation of UTI. Pro-inflammatory cytokine responses in the kidneys of androgenized mice were not apparent until 7 dpi, at which point renal abscesses had already been nucleated (21). Of note, our cytokine data were obtained from whole-kidney protein extracts, possibly insensitive to cytokine production that may be very localized near intrarenal foci of UPEC at earlier time points in infection (such as in the kidneys of vehicle-treated mice).

In other model systems, aged neutrophils are more responsive to infection than young neutrophils and have greater phagocytic capacity (44). Meanwhile, immature neutrophils are immunostimulatory but have reduced granular and phagocytic function compared to their mature counterparts (35, 36). Although these states have been investigated separately, prior studies have not examined the combined and orthogonal effects of both age and maturation. Here, we found that androgen exposure decoupled neutrophil aging from maturation within the infected urinary tract, as evidenced by the persistence of aged immature neutrophils in the androgenized kidney that comprised up to 40% of the total neutrophil population over the course of UTI. These neutrophils may have entered the bladder and kidneys as young immature cells, subsequently aging within the tissue milieu but not receiving, or properly responding to, maturation signaling.

This maturation defect was recapitulated in C57BL/6 mice, though there were fewer aged kidney neutrophils in all tested genotypes within this background. Neutrophil maturation was restored in LysM-Cre × AR^f/f^ C57BL/6 mice, but not in Ksp-Cre × AR^f/f^ mice, indicating that androgen-dependent maturation impairment predominantly reflects myeloid cell-intrinsic AR signaling, rather than broad effects exerted by androgenized kidney tissue. Of note, restoring maturation in LysM-Cre × AR^f/f^ mice did not alter kidney bacterial burden 7 dpi, indicating that androgen-dependent susceptibility to persistent high-titer pyelonephritis is not exclusively attributable to myeloid cell AR signaling. For example, previous studies have shown that androgens influence UTI susceptibility through priming renal epithelial cells for an aberrant wound healing response, induction of pro-fibrotic macrophage polarization, and a reduction in IL-17 producing γδ T-cells (17, 22, 49). AR-independent effects on UTI susceptibility and outcomes are also possible. In total, our results suggest that testosterone-dependent susceptibility to severe pyelonephritis reflects an array of effects on multiple cell types.

However, the maturation defect of neutrophils in the kidneys of androgen-exposed mice is not the only reason for their failure to control infection despite robust recruitment, as neutrophils that have trafficked specifically to the kidney have exhibited diminished antimicrobial functions. The degranulation response and phagocytic capacity of neutrophils (considered in aggregate) in the kidneys of androgenized mice were blunted and delayed, with granule release significantly reduced compared to vehicle-treated controls until 10 dpi. Interestingly, while the bladders of androgenized mice harbored an increased proportion of aged immature neutrophils 1 dpi compared to vehicle-treated mice, they had normal antimicrobial responses compared to vehicle-treated mice; only kidney neutrophils in androgenized mice exhibited dampened secretory vesicle secretion and reduced phagocytic capacity.

In an apparent effort to get the infection under control in the androgen-exposed host, mature neutrophils released primary granules 10 dpi, but by this time KBCs and foci of abscess have been established for several days (21). All neutrophil age/maturity subtypes isolated from the kidneys of naïve androgenized mice had lower phagocytic activity compared to those of vehicle-treated mice, and this effect was even more pronounced in neutrophils isolated from kidneys 7 dpi. Notably, though aged immature neutrophils reach UPEC within the tubular lumen, and therefore experience the same local milieu (e.g., cytokines) as other subtypes, these aged immature cells offered minimal primary degranulation and contributed little to phagocytosis within the KBC. These data indicate that while androgen exposure affects the maturation ability of tissue-infiltrated neutrophils, the kidney-specific environment in these mice exerts a compounding effect by further reducing neutrophil antimicrobial ability.

Sex differences occur in a variety of different infections and diseases, impacting susceptibility, immunity, and responses to treatment (23, 54, 57). We previously demonstrated that testosterone exposure enables persistent high-titer pyelonephritis and renal abscess formation (16, 21). We now identify a novel cellular basis for this observation, namely a tissue environment-specific effect of androgens on neutrophil maturation accompanied by a decrement in neutrophil function that is unique to the kidney. As our model infections focused on the urinary tract and analyses included blood, bone marrow, and spleen, it is intriguing to wonder whether other end organs, during bacterial infection, might also demonstrate analogous sex-discrepant effects on neutrophil maturation and function.

## MATERIALS AND METHODS

### Animals

All animals were group housed in temperature-controlled suites under timed light cycles. They were supplied standard mouse chow and water *ad libitum*. A minimum of two mice (and not exceeding five) were kept in cages with bedding and Nestlets. As indicated, most experiments were conducted in female C3H/HeN mice (#040, Envigo, Indianapolis, IN; RRID:MGI:2160972). Mice were androgenized as described previously (49) via weekly intramuscular injection of 150 mg/kg testosterone cypionate (Depo-Testosterone, Pfizer, New York, NY) beginning at 5 weeks of age and continuing until sacrifice; control animals were similarly injected with cottonseed oil. No animals were excluded from the analyses.

### Mouse strain creation

Background strains for AR knockouts were originally purchased from Jackson Laboratories (Bar Harbor, ME). Ksp-Cre × AR^f/f^ mice were generated by crossing B6.129S1-*Ar^tm2.1Reb^*/J (#018450) mice with B6.Cg-Tg(Cdh16-cre)91Igr/J (#012237) to create mice homozygous for floxed *Ar* and hemizygous for the *cre* recombinase gene under the control of the cadherin 16 (kidney-specific cadherin [Ksp]) promoter. LysM-Cre × AR^f/f^ mice were similarly generated by crossing the same homozygous floxed *Ar* mice with C57BL/6 mice expressing *cre* under the LysM promoter (kind gift from S. C. Morley). For the experiments using these strains, mice for the vehicle-treated and androgenized Cre^–^AR^f/f^ control groups were randomly chosen littermates from both Ksp-Cre × AR^f/f^ and LysM-Cre × AR^f/f^ breeders.

### Bacterial strains

UTI89, a clinical isolate of uropathogenic *E. coli* (UPEC) (58), was grown statically at 37°C in Luria-Bertani broth (LB; Becton Dickinson, Sparks, MD). Overnight cultures were centrifuged at 7,500 × *g* at 4°C, and the resulting pellet was resuspended in sterile phosphate-buffered saline (PBS) to a final density of ~4 × 10^8^ colony-forming units (CFU)/mL. UTI was initiated in the morning by transurethral inoculation of 50 µL of prepared bacterial suspension, delivering an inoculum of 1–2 × 10^7^ CFU. For *ex vivo* experiments, the chromosomally GFP-expressing strain UTI89 *att*_HK022_::COM-GFP was used (59), and heat killing was performed at 60°C for 30 min.

### Determination of bacterial loads

At the indicated time points, mice were anesthetized approximately 1 h into their light cycle with inhaled isoflurane (Patterson Veterinary, Loveland, CO), and terminally perfused with 4°C PBS through the left ventricle. Bladders and kidneys were aseptically removed and homogenized into sterile PBS before serial dilution and plating on Luria-Bertani agar.

### Flow cytometry

Mice were sacrificed as described above, and harvested kidneys were manually homogenized through a 70-µm cell strainer into cold RPMI (Gibco), then centrifuged for 5 min (500 *× g*) at 4°C. The resulting pellets were resuspended in room-temperature RBC lysis buffer (155 mM NH_4_Cl, 10 mM KHCO_3_), washed with cold fluorescence-activated cell sorting (FACS) buffer (10% fetal bovine serum [FBS], 1% [wt/vol] sodium azide in PBS), and subjected to a Percoll gradient to enrich for leukocytes. For the gradient, cell pellets were resuspended in a solution containing 36% (vol/vol) Percoll PLUS (Cytivia, Uppsala, Sweden), 25 mM sucrose in PBS, and layered on top of a solution containing 72% (vol/vol) Percoll PLUS, 25 mM sucrose in FACS buffer. Gradients were centrifuged (500 *× g*) for 30 min at 4°C, and enriched leukocytes were collected from the buffy coat. For peripheral blood analysis, blood was collected into K2 EDTA collection tubes (BD Vacutainer #366643) via cardiac puncture before perfusion. Bone marrow was isolated from femurs. Peripheral blood, spleen, and bone marrow leukocytes were subjected to RBC lysis but did not undergo Percoll separation. Bladders were quadrisected and washed gently three times in sterile PBS to remove leukocytes from the urinary space. Washed bladders were incubated for 1 h at 37°C in 0.34 U/mL of Liberase (Roche) in PBS. Digestion was halted by the addition of FACS buffer, and the digested bladders were passed through a 70-µm cell strainer before staining. Bladder cell preparations were not subjected to hypotonic lysis or Percoll separation.

Isolated leukocytes were stained with Live/Dead Fixable Blue (ThermoFisher Scientific) in PBS, washed, and blocked with Fc Block (BD Biosciences, San José, CA) before staining with the extracellular antibodies listed in Table S1 in FACS buffer. Cells were washed, resuspended in FACS buffer, and analyzed with an Aurora flow cytometer (Cytek Biosciences, Fremont, CA) and FlowJo software (BD Biosciences). A representative gating scheme is provided in Fig. S6.

### Tissue preparation and immunofluorescence

Mice were sacrificed as described, and harvested kidneys were placed in 4% paraformaldehyde in PBS at 4°C for 1 h before being transferred to sterile 30% sucrose overnight at 4°C. Kidneys were embedded and frozen in OCT (Fisher Scientific, Hampton, NH); blocks were then cryosectioned into 5–8 µm sections and mounted on Superfrost Plus slides (Fisher Scientific).

For immunofluorescence staining, sections were rinsed with PBS to remove OCT, permeabilized with 0.25% Triton X-100 (Sigma) in PBS for 10 min, blocked for 1 h with 10% FBS (Gibco) in PBS, then stained with the primary antibodies listed in Table S2 in the blocking buffer. Slides were washed three times with PBS before staining with the secondary antibody listed in Table S2 in the blocking buffer. Stained slides were washed again, mounted with Prolong Gold Antifade Reagent (ThermoFisher Scientific #P36930), and imaged with a Zeiss LSM 880 Airyscan confocal microscope (Oberkochen, Germany).

### *Ex vivo* phagocytosis assay

Kidneys were harvested and prepared for flow cytometry as described above. After the Percoll gradient, 1 × 10^6^ cells (by hemacytometer) were incubated with 1 × 10^6^ CFU of UTI89 *att*_HK022_::COM-GFP (59) in RPMI 1640 (Gibco) + 10% FBS for 30 min at 37°C with 5% CO_2_. Cells were then washed with FACS buffer, and staining was performed as described in the preceding section. The gating of GFP+ UPEC is shown in Fig. S6.

### Protein extraction

Harvested kidneys were flash-frozen in liquid nitrogen and stored at −80°C until use. Kidneys were homogenized in radioimmunoprecipitation assay (RIPA) buffer (50 mM Tris-HCl, 150 mM NaCl, 1% [vol/vol] Nonidet P-40, 0.1% [wt/vol] SDS, 0.5% [wt/vol] sodium deoxycholate, pH 7.4) containing PhosSTOP phosphatase inhibitor and cOmplete Mini protease inhibitor (Roche; Basel, Switzerland). Lysates were cleared by centrifugation (2 × 5 min in a tabletop microcentrifuge at maximum speed), and protein concentration was determined by BCA assay (Invitrogen, Carlsbad, CA).

### Cytokine quantification

Extracted whole-kidney protein was diluted in PBS to 900 µg/mL and subjected to a Bio-Plex Mouse Cytokine 23-plex Assay (#M60009RDPD, Bio-Rad, Hercules, CA), according to the manufacturer instructions. The plate was read with a Bio-Plex 200 system and analyzed using BioPlex Manager 6.1 software.

### RNA extraction and qPCR

RNA was isolated from kidneys and bone marrow using RNA STAT-60 (TEL-TEST; Friendswood, TX), according to the manufacturer’s instructions. RNA was converted to cDNA with the iScript cDNA synthesis kit (Bio-Rad) before qPCR analysis. qPCR was done using iTaq Universal SYBR Green Supermix (Bio-Rad), containing 350 nM primers and 20 ng of cDNA per reaction, and thermal cycling was performed on a 7500 Fast RT-PCR system (Applied Biosystems, Foster City, CA) as follows: 95°C, 3 min, 40× (95°C 10 s, 60°C, 30 s). *Gapdh* was used as the kidney housekeeping gene (F: 5′ GATGCTGCCCTTACCCCGG 3′, R: 5′ CAAATGGCAGCCCTGGTGACC 3′), and *Ppid* was used for bone marrow (F: 5′ ATGGTGAAAAACCTGCCAAA 3′, R: 5′ CATCCTCAGGGAAGTCTGGA 3’). *Ar* was probed in both organs with the following primers: F: 5′ CCTTGGATGGAGAACTACTCCG 3′, R: 5′ TCCGTAGTGACAGCCAGAAGCT 3′. Samples with undetected Ct values were calculated based on a limit of detection of Ct = 40.

### Statistical analysis

Statistical analyses were performed by unpaired, non-parametric Mann-Whitney U tests using GraphPad Prism 9.5.0. *P* values < 0.05 were considered significant. Statistical details for each figure can be found in the corresponding figure legend.

## SUPPLEMENTAL MATERIAL

Supplemental Tables and Figures mbio.03170-23-s0001.pdf Tables S1 and S2 and Fig. S1–S6.

ASM does not own the copyrights to Supplemental Material that may be linked to, or accessed through, an article. The authors have granted ASM a non-exclusive, world-wide license to publish the Supplemental Material files. Please contact the corresponding author directly for reuse.
