# Supplementary material for: Androgen exposure impairs neutrophil maturation and function within the infected kidney
Source: mBio. 2024 Jan 11;15(2):e03170-23. doi: 10.1128/mbio.03170-23 (PMC10865792; doi:10.1128/mbio.03170-23)
Supplement: Supplemental Tables and Figures — Tables S1 and S2 and Fig. S1–S6. [file mbio.03170-23-s0001.pdf]

Supplemental Material

Androgen exposure impairs neutrophil maturation and function within the infected kidney

Teri N. Hreha,[1] Christina A. Collins,[1] Elisabeth B. Cole,[1] Rachel J. Jin,[1] and David A. Hunstad[1,2]

Departments of [1]Pediatrics and [2]Molecular Microbiology, Washington University School of

Medicine, St. Louis, Missouri, USA

ANTIBODY	DILUTION	SOURCE	IDENTIFIER
Rat anti-mouse MHCII (2G9) BUV395	1:400	BD Optibuild	Cat#743876; RRID: AB_2741827
Rat anti-mouse CD19 (1D3) BUV661	1:200	BD Horizon	Cat#612971; RRID: AB_2870243
Rat anti-mouse CD45 (30-F11) BV510	1:400	BD Pharmingen	Cat#563891; RRID: AB_2734134
Rat anti-mouse/human CD11b (M1/70) BV570	1:400	Biolegend	Cat#101233; RRID: AB_10896949
Rat anti-mouse CD18 (M18/2) BV650	1:200	BD Optibuild	Cat#744600; RRID: AB_2742349
Rat anti-mouse CD49d (R1-2) BV711	1:50	BD Optibuild	Cat#740661; RRID: AB_2740350
Armenian hamster anti-mouse CD11c (N418) BV785	1:200	Biolegend	Cat#117336; RRID: AB_2565268
Rat anti-mouse CD3ε (17A2) SparkBlue 550	1:200	Biolegend	Cat#100260; RRID: AB_2832258
Rat anti-mouse F4/80 (T45-2342) BB700	1:200	BD Optibuild	Cat#746070; RRID: AB_2743450
Rat anti-mouse CD49b (DX5) PE/Dazzle 594 (for C3H/HeN mice)	1:50	Biolegend	Cat#108924; RRID: AB_2565271
Mouse anti-mouse NK1.1 (PK136) PE/Dazzle 594 (for C57BL/6 mice)	1:400	Biolegend	Cat#108748; RRID: AB_2564219
Rat anti-mouse Ly6C (ER-MP20) PE/Cy5.5	1:200	Novus	#NB100-65413PECY55; RRID: AB_2136654
Rat anti-mouse CD101 (307707) Alexa Fluor 647	1:100	BD Pharmingen	Cat#564473; RRID: AB_2738821
Rat anti-mouse Ly6G (1A8) Alexa Fluor 700	1:800	Biolegend	Cat#127621; RRID: AB_10640452
Rat anti-mouse CD63 (NVG-2) APC/Cy7	1:200	Biolegend	Cat#143908; RRID: AB_2565498
Rat anti-mouse CXCR4 (2B11) BUV805	1:200	BD Optibuild	Cat#741979; RRID: AB_2871282
Rat anti-mouse CD62L (MEL-14) PerCP	1:100	Biolegend	Cat#104430; RRID: AB_2187124
Rat anti-mouse CXCR2 (SA044G4) PE/Cy7	1:200	Biolegend	Cat#149316; RRID: AB_2734213

Table S1. Antibodies used for flow cytometry.

PRIMARY ANTIBODY	DILUTION	SOURCE	IDENTIFIER
Goat anti-*E. coli*, O and K serotypes	1:200	Meridian Life Science	Cat#B65109G; RRID: AB_151291
Rat anti-mouse Ly6G (1A8) APC	1:200	Biolegend	Cat#127614; RRID: AB_2227348
Rat anti-mouse CD101 (Moushi101) PE	1:150	eBiosciences	Cat#12-1011-82; RRID: AB_1210728
Rat anti-mouse CD49d (R1-2) Alexa Fluor 488	1:150	Biolegend	Cat#103611; RRID: AB_528834
SECONDARY ANTIBODY	DILUTION	SOURCE	IDENTIFIER
Donkey anti-goat-Alexa Fluor 594	1:200	Abcam	Cat#ab150132; RRID: AB_2810222

Table S2. Antibodies used for immunofluorescence staining.

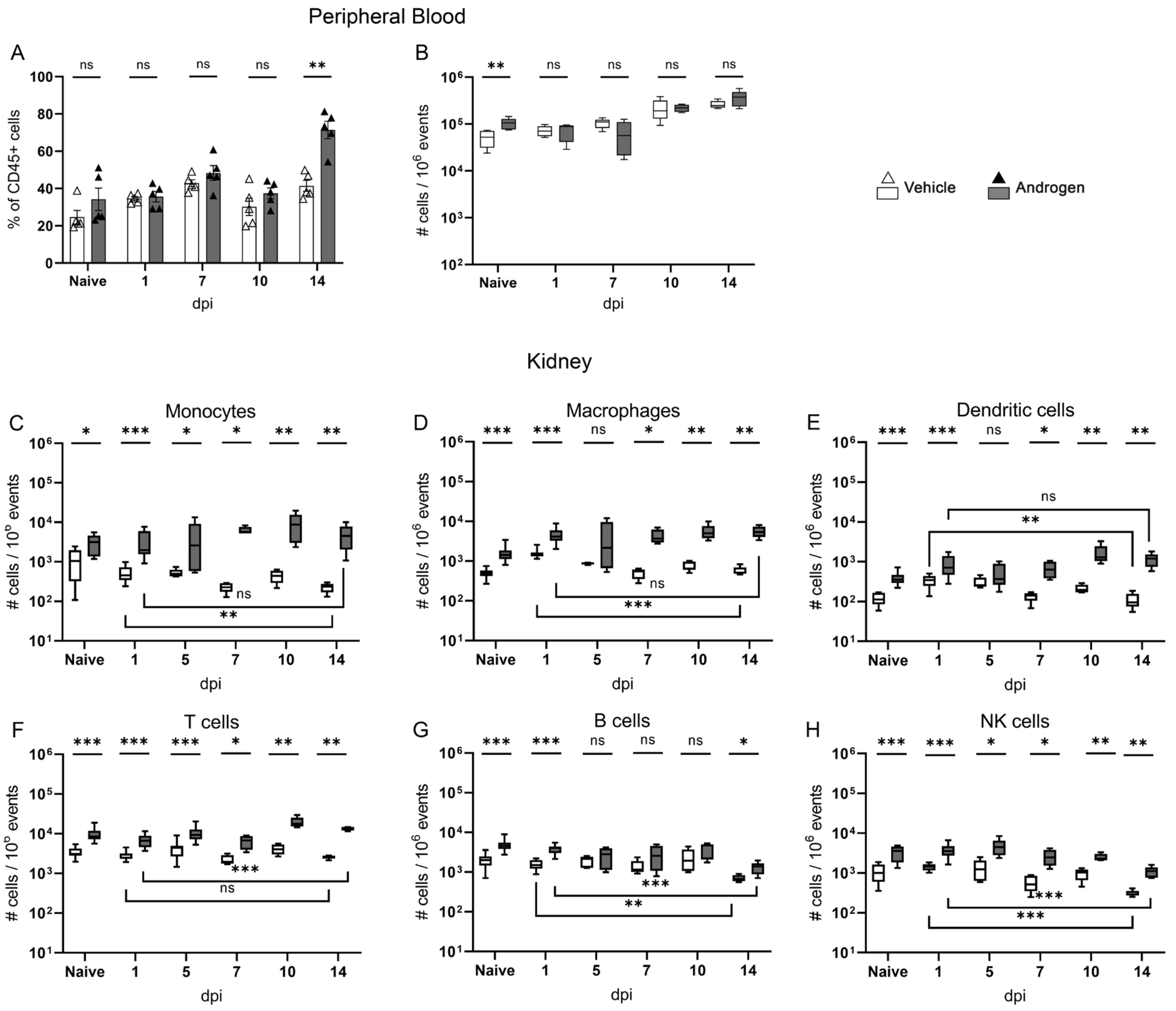

Figure S1. Timeline of the neutrophil population in peripheral blood and leukocyte populations in the kidneys. The population of neutrophils in the peripheral blood, as a proportion of CD45+ cells (A) and absolute count (per million events; B) are shown for vehicle-treated (open triangles, white bars) and androgenized mice (closed triangles, gray bars). (C-H) Leukocyte subset populations over time in the kidneys of vehicle-treated (white bars) and androgenized mice (gray bars). Bars indicate mean with SEM, Box and whisker plots represent 95 percentile range with min and max. Each symbol represents a single mouse; n = 5-15 per time point. *$P < 0.05$, **$P < 0.01$, ***$P < 0.001$ by Mann-Whitney U test. ns, not significant.

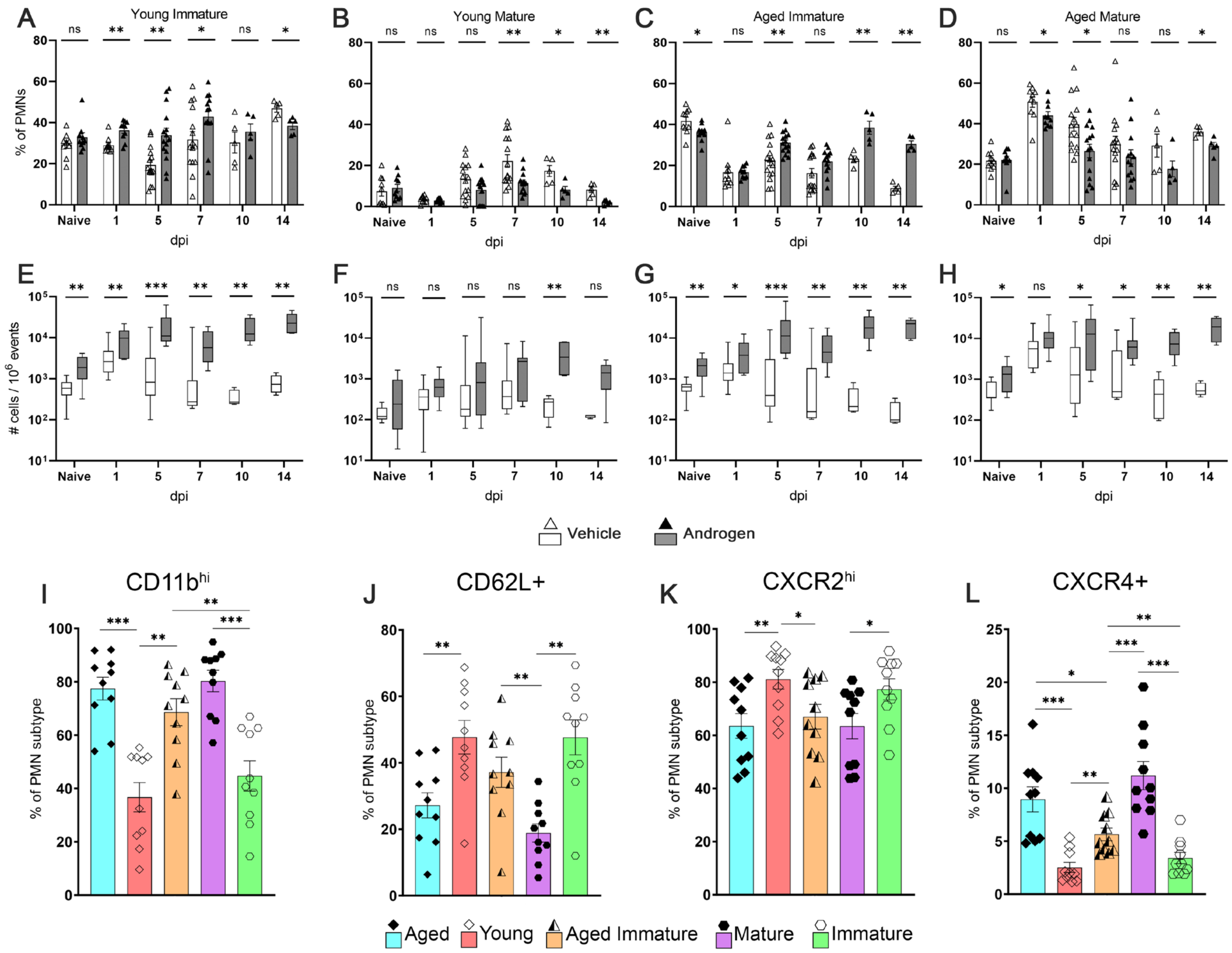

Figure S2. Populations of neutrophil age and maturity subtypes in the kidneys throughout infection. Relative proportion of all neutrophils (PMNs; A-D) and absolute number of neutrophils (per million events; E-H), as measured by flow cytometry, represented by young immature (A, E), young mature (B, F), aged immature (C, G), or aged mature (D, H) subtypes, in the kidneys of vehicle-treated (open triangles, white bars) or androgenized mice (closed triangles, gray bars). (I-L) Percent of neutrophils characterized as CD11b^{hi} (I), CD62L+ (J), CXCR2^{hi} (K) and CXCR4+ (L) in a representative sample of neutrophils from ice infected for 5 or 7 days, gated solely on maturity (purple, filled hexagons; mature, green, open hexagons; immature) or age (blue, filled diamonds; aged, red, open diamonds; young), compared to aged immature neutrophils (orange, half-filled triangles). Bars indicate mean with SEM. Box and whisker plots represent 95 percentile range with min and max. Each symbol represents a single mouse; n = 5-15 mice per time point. **P* < 0.05, ***P* < 0.01 by Mann-Whitney U test. ns, not significant.

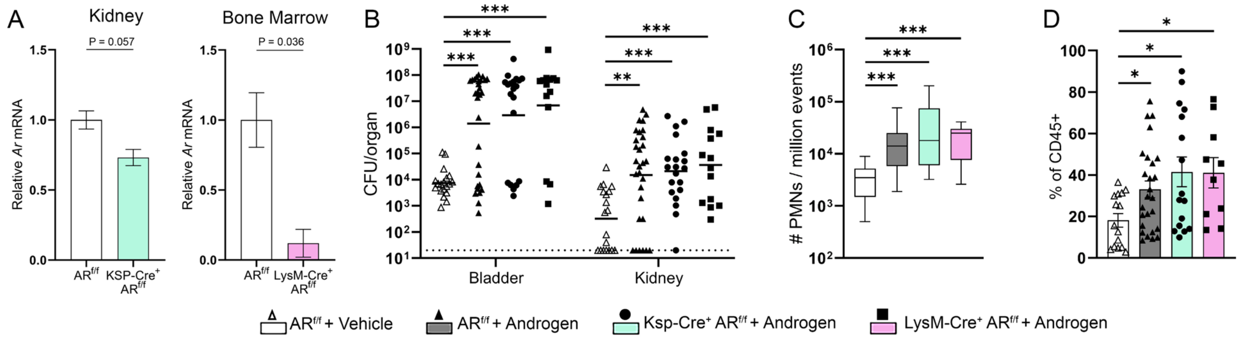

Figure S3. Androgenized C57BL/6 mice exhibit chronic high-titer pyelonephritis, even with conditional AR deficiencies. (A) qPCR measurement of androgen receptor (*Ar*) mRNA in the kidneys of Ksp-Cre × $AR^{f/f}$ mice (green bars) and bone marrow of LysM-Cre × $AR^{f/f}$ mice (pink bars) compared to Cre^{-}$AR^{f/f}$ litterlate controls (white bars). n = 3-5 mice per group. (B) Bladder and kidney bacterial loads in vehicle-treated Cre^{-}$AR^{f/f}$ (open triangles, white bars), androgenized Cre^{-}$AR^{f/f}$ (closed triangles, gray bars), androgenized Ksp-Cre × $AR^{f/f}$ (squares, green bars), or androgenized LysM-Cre × $AR^{f/f}$ (circles, pink bars) mice 7 days post infection. Lines indicate geometric mean. (C) Kidney neutrophil (PMN) counts in the same groups of mice (per million events). Box and whisker plots represent 95 percentile range with min and max. (D) Kidney neutrophils in the same groups of mice as a percentage of CD45+ cells. Bars indicate mean with SEM. n = 15-26 mice per group; *$P < 0.05$, * $P < 0.01$, ***$P < 0.001$ by Mann-Whitney U test. CFU, colony-forming units.

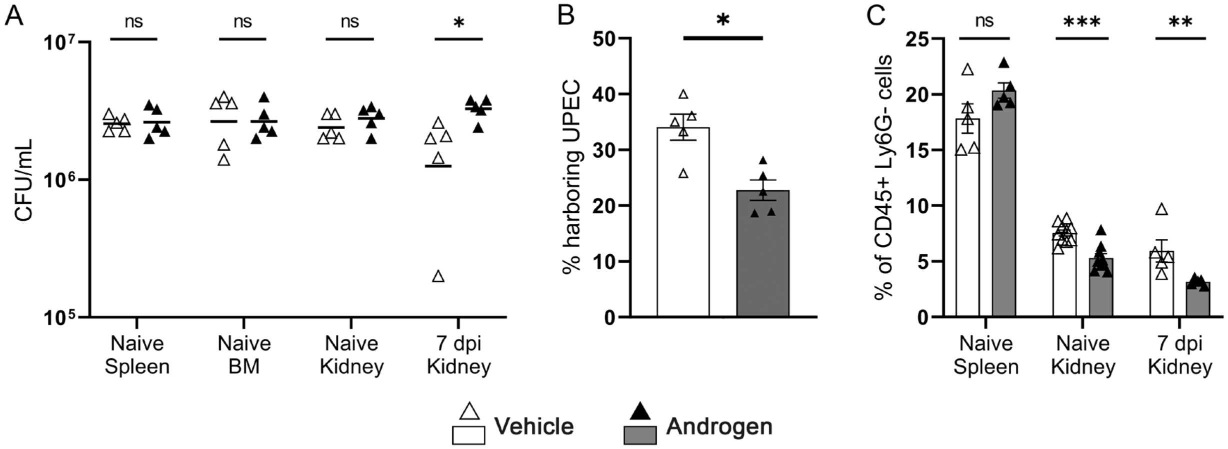

Figure S4. Remaining UPEC after *ex vivo* phagocytosis assay and phagocytic capacity of non-neutrophil leukocytes in C3H/HeN kidneys. (A) Bacterial colony-forming units (CFU) of GFP+ UPEC in supernatants after 30 min of exposure to non-neutrophil leukocytes (CD45+ Ly6G–) isolated from naïve spleen, bone marrow (BM), or kidneys, or from kidneys 7 days post infection (dpi) with UPEC, in vehicle-treated (open triangles) or androgenized mice (closed triangles). Lines indicate geometric mean. (B) Phagocytic capacity (as measured by % GFP positive) of neutrophils isolated from kidneys of naïve mice treated with vehicle (white bars) or androgen (gray bars) when exposed to heat-killed UPEC. (C) Phagocytic capacity of CD45+ Ly6G– leukocytes isolated from naïve spleen or kidneys, or from kidneys 7 dpi, of mice treated with vehicle or androgen. Bars indicate mean with SEM. n = 5-10 mice per group; $^{**}P < 0.01$, $^{***}P < 0.001$ by Mann-Whitney U test. ns, not significant.

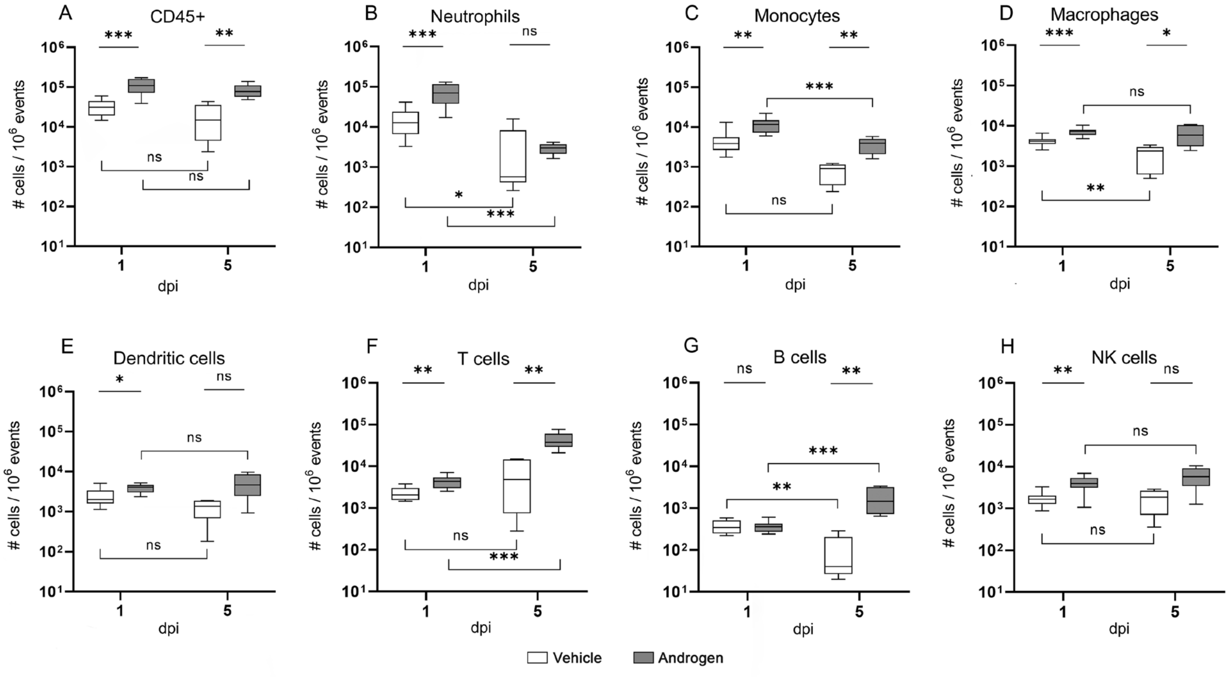

Figure S5. Timeline of leukocyte populations in the bladder. (A-H) Absolute count per million events, in vehicle-treated (white boxes) and androgenized mice (gray boxes), of all leukocytes (A) and leukocyte subset populations (B-H) in the bladder 1 and 5 dpi. Box and whisker plots represent 95 percentile range with min and max. n = 5-10 per time point. *$P < 0.05$, **$P < 0.01$, ***$P < 0.001$ by Mann-Whitney U test. ns, not significant.

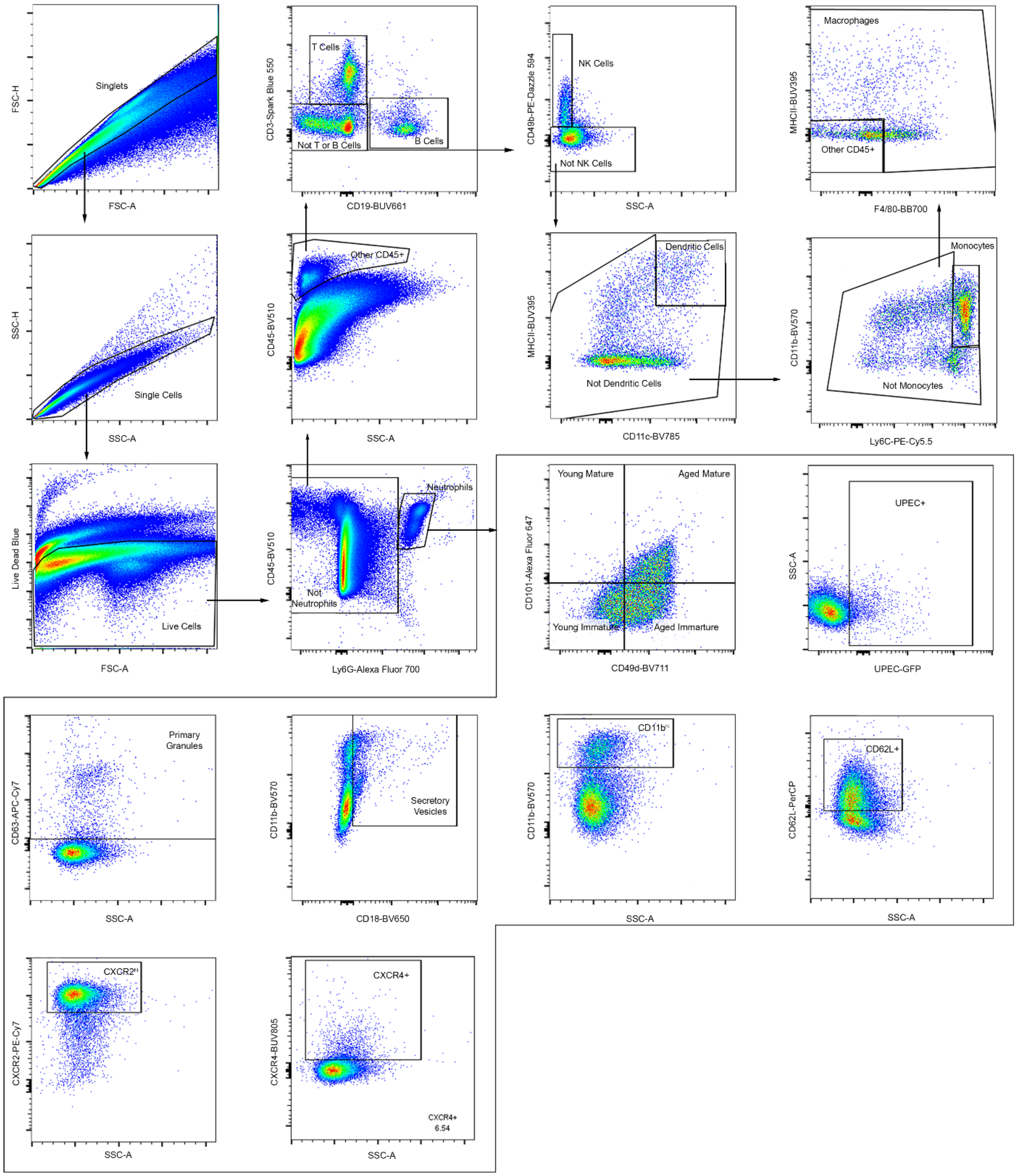

Figure S6. Representative gating scheme for analyzing leukocytes and neutrophils from mouse kidneys.
